# Mechanical Performance of Unstitched and Silk Fiber-Stitched Woven Kenaf Fiber-Reinforced Epoxy Composites

**DOI:** 10.3390/ma13214801

**Published:** 2020-10-28

**Authors:** Yasir Khaleel Kirmasha, Mohaiman J. Sharba, Zulkiflle Leman, Mohamed Thariq Hameed Sultan

**Affiliations:** 1 Department of Mechanical and Manufacturing Engineering, Faculty of Engineering, Universiti Putra Malaysia, Serdang 43400, Malaysia; zleman@upm.edu.my; 2 Power Mechanics Department, AL-Suwayrah Technical Institute, Middle Technical University, Baghdad 10074, Iraq; mohaimaneng@gmail.com; 3 Advanced Engineering Materials and Composites Research Centre, Faculty of Engineering, Universiti Putra Malaysia, Serdang 43400, Malaysia; 4 Department of Aerospace Engineering, Faculty of Engineering, Universiti Putra Malaysia, Serdang 43400, Malaysia; thariq@upm.edu.my; 5 Laboratory of Biocomposite Technology, Institute of Tropical Forestry and Forest Products (INTROP), Universiti Putra Malaysia, Serdang 43400, Malaysia; 6 Aerospace Malaysia Innovation Centre (944751-A), Prime Minister’s Department, MIGHT Partnership Hub, Jalan Impact, Selangor Darul Ehsan 43400, Malaysia; 7 UPM Press, Universiti Putra Malaysia, UPM Serdang 43400, Malaysia

**Keywords:** textiles, stitching, kenaf, natural fibers, through-thickness mechanical properties

## Abstract

Fiber composites are known to have poor through-thickness mechanical properties due to the absence of a Z-direction binder. This issue is more critical with the use of natural fibers due to their low strength compared to synthetic fibers. Stitching is a through-thickness toughening method that is used to introduce fibers in the Z-direction, which will result in better through-thickness mechanical properties. This research was carried out to determine the mechanical properties of unstitched and silk fiber-stitched woven kenaf-reinforced epoxy composites. The woven kenaf mat was stitched with silk fiber using a commercial sewing machine. The specimens were fabricated using a hand lay-up method. Three specimens were fabricated, one unstitched and two silk-stitched with deferent stitching orientations. The results show that the stitched specimens have comparable in-plane mechanical properties to the unstitched specimens. For the tensile mechanical test, stitched specimens show similar and 17.1% higher tensile strength compared to the unstitched specimens. The flexural mechanical test results show around a 9% decrease in the flexural strength for the stitched specimens. On the other hand, the Izod impact mechanical test results show a significant improvement of 33% for the stitched specimens, which means that stitching has successfully improved the out-of-plane mechanical properties. The outcome of this research indicates that the stitched specimens have better mechanical performance compared to the unstitched specimens and that the decrease in the flexural strength is insignificant in contrast with the remarkable enhancement in the impact strength.

## 1. Introduction

Over the last decade, natural fibers have been more commonly used as composite resin-enforcements in fiber-reinforced polymer composites due to their good mechanical properties, low cost, high specific strength and, above all, their eco-friendly and biodegradability characteristics [1,2,3]. Natural fibers are widely used as reinforcements for polymer composites instead of glass fiber and other synthetic fibers in many applications, especially in sports goods and transportation industries [4,5,6]. In transportation industries, natural fibers are being used to fabricate many car components such as door panels, car roofs, dashboards components, mats and wheels [7]. In sports goods, many types of equipment, like surfboards, windsurfing equipment, tennis rackets, badminton rackets and golf clubs, are now being fabricated using natural fibers [8]. In addition, natural fibers are also being used as packaging materials, such as environmentally friendly bags, ropes and carpets [9,10].

Among natural fibers, kenaf fiber or (*hibiscus cannabinus*) has been increasingly used as reinforcement in composites in recent years. Kenaf fiber is considered as attractive plant fiber because of its high growth rate at different climates and its low cost [11]. While there are many advantages to using natural fibers, there are also some serious drawbacks, such as their high moisture absorption and poor wettability, which result in poor bonding between the natural fiber and the polymer matrix due to the difference between the materials’ densities [12]. These drawbacks have their impact on the use of natural fibers in the skeleton structure applications and aerospace sectors [13].

In general, laminated fiber-reinforced polymer (FRP) composites show poor mechanical performance in the out-of-plane direction. This poor mechanical performance results from the missing Z-direction fibers [14]. The out-of-plane properties of the composite, like impact properties, have been one of the most critical design considerations as laminate composites are known to suffer from low impact properties. This behavior is more crucial when natural fibers are used as reinforcing fibers for the composite [15]. Therefore, many three-dimensional (3D) methods have been developed, such as 3D braiding, weaving, knitting and stitching. These methods work to introduce through-thickness fibers (Z-direction fibers), which work to increase the delamination resistance of the laminate system [16,17]. Among the 3D toughening methods, stitching is a unique method because it is simple, low in cost and works effectively.

The stitching method started to receive increasing attention in the mid-1980s, when a through-thickness fiber or yarn was used to reinforce polymer composites. The attention was aroused because the majority of (2D) fiber reinforced polymer composites are well known to have poor through-thickness mechanical properties. In this method, two or more layers of 2D fabric are stitched together by a needle that carries Z-directional yarn through the layers, creating a 3D architecture. This method allows us to use different types of materials together and with different yarn directions. Moreover, this method can be used to create 3D complicated architectures by stitching different parts together. However, this method causes degradation in the in-plane mechanical properties and causes local fiber damage, resin pockets and crimps to the stitched fiber [18].

Stitching has proven to be a well-applied method to improve both the low-velocity impact [19,20] and high-velocity impact [21,22] strength of synthetic fiber composites. In addition, it was reported by Bhudolia et al. [23] that both energy absorption and load peak were improved after stitching the carbon fiber-reinforced epoxy composite. However, the effect of the through-thickness stitching on the in-plane and through-thickness mechanical properties of the natural fiber-reinforced polymer composites has not been extensively investigated, as is the case for synthetic fiber-reinforced polymer composites [15,24].

Mohd Yuhazri Yaakob et al. [25] studied the effect of stitching patterns on the tensile strength of kenaf woven fabric composites. In their study, the woven kenaf was stitched with two different patterns, which were the single-stitched pattern and double-cross pattern. It was found that the increase in the specific strength for the single-stitched pattern composite was up to 14.51% compared to the unstitched composite. On the other hand, the double-stitched pattern composite showed a 53.17% improvement in the specific strength compared to the unstitched composite. It was concluded that the double-stitched pattern composite showed the best mechanical performance and that stitching patterns and stitching angle play a great role in the determination of the mechanical performance of woven kenaf composite.

Rong et al. [24] investigated the effect of stitching on the tensile properties, flexural properties and Mode I interlaminar fracture toughness of stitched unidirectional sisal-reinforced epoxy laminates. Nylon, Kevlar and sisal threads were used for the through-thickness stitching. It was found that both the tensile and flexural properties of the laminates were not considerably affected by the through-thickness stitching, while the interlaminar toughness was much improved. It was also found that sisal laminates have a slightly higher tolerance to stitching damages compared to glass fiber-reinforced polymer composites.

Ravandi et al. [26] investigated the effect of the through-thickness stitching areal fraction on the Mode I interlaminar fracture and the in-plane tensile behavior of flax-reinforced epoxy laminates stitched by flax and cotton threads. It was found that stitching reduced the tensile properties of both stitched laminates due to the imperfections caused by stitching. It was also shown that stitching with cotton thread did not increase the interlaminar fracture toughness of the laminate. However, stitching with low fiber areal fraction using flax thread increased the interlaminar fracture toughness by around 10%.

Another study involving natural fiber was carried out by [15]. In their work, they investigated the effect of the through-thickness stitching on the low-velocity impact response of the woven flax-reinforced epoxy laminates stitched by flax and cotton threads. It was found that delamination was not the main damage mode in either unstitched or stitched laminates. However, stitching works to facilitate the propagation of in-plane cracks. It was also found that stitching with thick thread resulted in a lower percentage of absorbed energy per area of damage.

Based on this, the composites in this study were fabricated using natural fiber materials, which are woven kenaf and silk yarn, for stitching to contribute to the field of natural fiber-stitched composites and also to understand the in-plane and through-thickness mechanical behaviors of stitched natural fibers. A better understanding of stitched natural fibers might lead to the expansion of their uses and applications in different fields and sectors.

This research aims to investigate the stitching effect on tensile strength, flexural strength and impact behavior of a woven fiber-reinforced thermoset composite fabricated using woven kenaf and epoxy resin. The effects of stitching thread, stitch row orientations and stitch row spacings on the in-plane and through-thickness mechanical performance were studied. Impact properties were detected using an instrumented Izod impact test. Silk thread was used as a sewing thread in this study.

## 2. Materials and Methods

### 2.1. Materials

**Figure 1:**
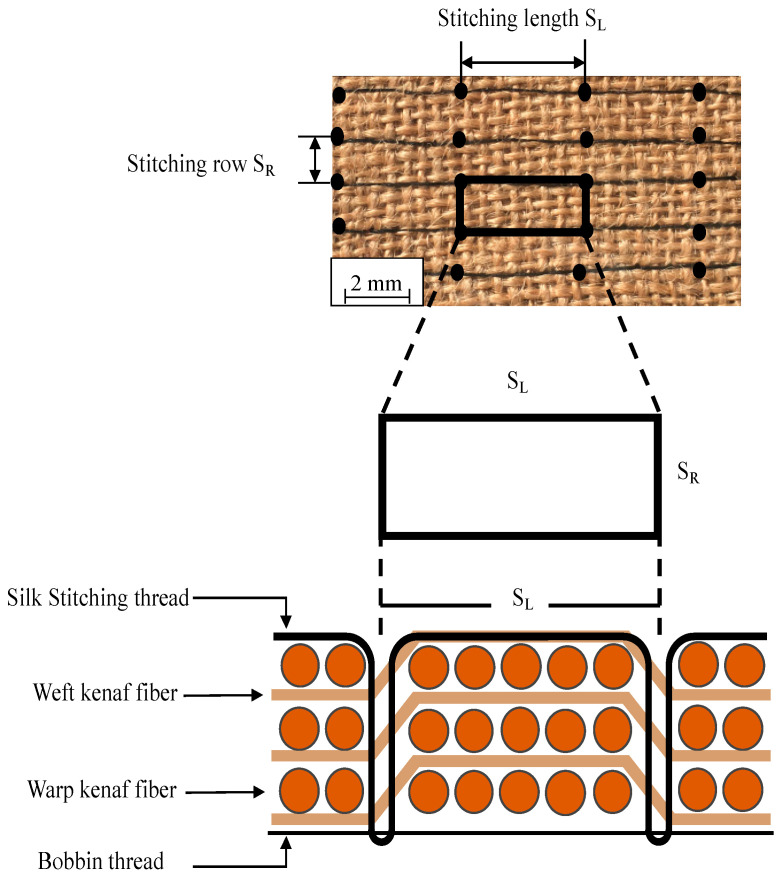
Woven kenaf stitching process.

The specimens were made using woven kenaf fiber (GO green, Chennai, India) as reinforcement material. The woven kenaf composite was fabricated using a lay-up of 3 layers. Silk thread (Gütermann, Breisgau, Germany) with a cross-sectional area of 0.06 mm^2^ was used as the through-thickness stitching material. Silk fiber was chosen as the stitching material not only because it comes from natural origin but also because silk fibers show higher mechanical properties than plant fibers. Silk fiber shows equivalent specific mechanical properties to glass fiber in some cases [27]. The woven kenaf stitching process was carried out using a typical sewing machine. The stitching process involves inserting silk thread through woven kenaf laminates to create a 3D structure. The stitching process parameters were S_L_ = 5 mm (distance between two needle insertion points in the stitching row) and S_R_ = 5 mm (distance between one stitching row and the other). The parameters of the stitching process are illustrated in Figure 1. Stitching density was calculated according to the following equation.

(1) $$SD=\;\frac{1}{S_{R\;}\times S_{L}}$$

Fiber volume fractions of unstitched and silk-fiber-stitched woven kenaf-reinforced epoxy composites were calculated using the pre-stated formula:

(2) vf%=[(wkρk)(wkρk)+(wmρm)]∗100 %

where $w_{k}$ and $w_{m}$ are kenaf weight fraction and matrix weight fraction, respectively. The density of kenaf is represented by $\rho_{k}$ and the density of the epoxy matrix is represented by $\rho_{m}$. The void content of all composites was neglected in this study. Stitching fibers were also taken into consideration in the calculation of fiber volume fraction. The average values of the fiber volume fractions of all composites are shown in Table 1.

**Figure 2:**
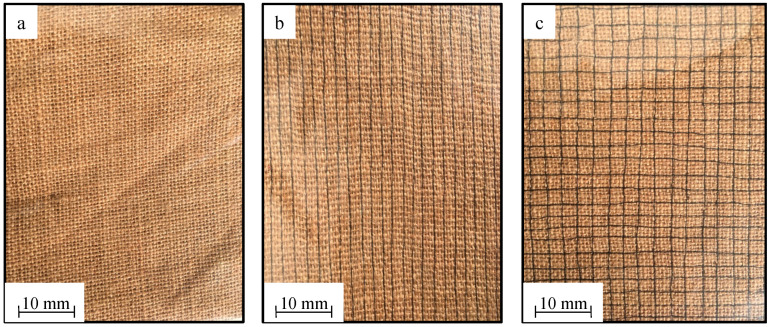
Unstitched and silk-fiber-stitched woven kenaf: (**a**) UNSTITCHED; (**b**) WKS/X; (**c**) WKS/XY.

Three specimens were fabricated in this study, one unstitched and two stitched specimens, as shown in Figure 2. The two stitched specimens have different stitching densities and orientations. The first stitched specimen is (WKS/X) which is woven kenaf stitched with silk-fiber in X-direction only. The second stitched specimen is (WKS/XY) which is woven kenaf stitched with silk-fiber in both X-direction and Y-direction. A summary of the stitching parameters and notation used in this study is given in Table 1.

### 2.2. Preparation of Composite Laminates

**Figure 3:**
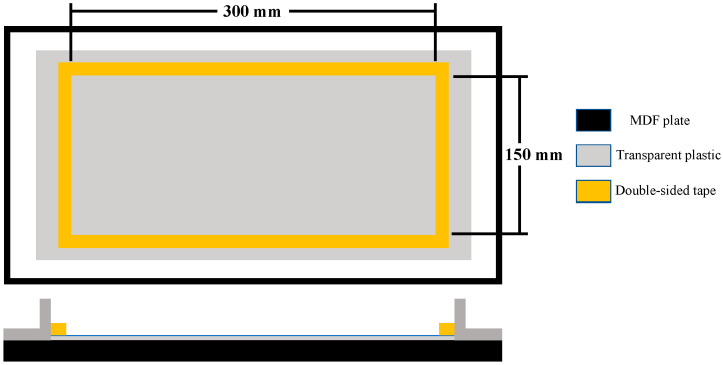
Schematic diagram of mold.

A hand lay-up method was used to fabricate the unstitched and stitched woven kenaf-reinforced epoxy composites. The dimensions of the mold that was used in this study are 300 mm in length and 150 mm in width. Double-sided tape was used to draw the dimension of the mold and a plastic film was used to prevent the sticking of the epoxy with the medium-density fiberboard (MDF) flat surface that was used for the fabrication, as shown in Figure 3. Three layers of woven kenaf with 300 mm length and 150 mm width were used for the fabrication process. The woven kenaf had the same number of warp yarns and weft yarns per unit area. Before fabrication, all the woven kenaf (unstitched and stitched) pieces were dried at 80° Celsius for 24 h using a ventilated oven. The average value of moister content was (7.3% of the total weight of the specimens). The percentage of the weight decrease was calculated by subtracting specimens’ weight after heating values from specimens’ weight before heating values divided by specimens’ weight before heating values then multiplying this by 100. After this, the average was calculated by adding the obtained results and dividing them by the number of specimens. An epoxy system (EpoxAmite^TM^ 100) with (102 medium) hardener supplied by (SATSUKU Corporation, Tokyo, Japan) was used with a mixing ratio of 100 Å: 24 B by weight according to the manufacturer’s recommendations. In the unstitched case, each layer of the fiber was impregnated in the mold individually, introducing resin between every single layer. However, for the stitched woven kenaf, two layers of resin were poured, one under the stitched woven preform directly onto the mold and the other at the top of it. A steel roller was used in both cases to ensure that the resin was distributed equally and also to remove air bubbles that formed during the fabrication process. After laying the composite inside the mold was completed, a flat surface plate was used to apply pressure on the composite. The applied pressure was (10 kg × 9.81 = 98.1 N). The composite was left to cure at room temperature for 24 h under the applied pressure.

### 2.3. Characterization

#### 2.3.1. Tensile Testing

**Figure 4:**
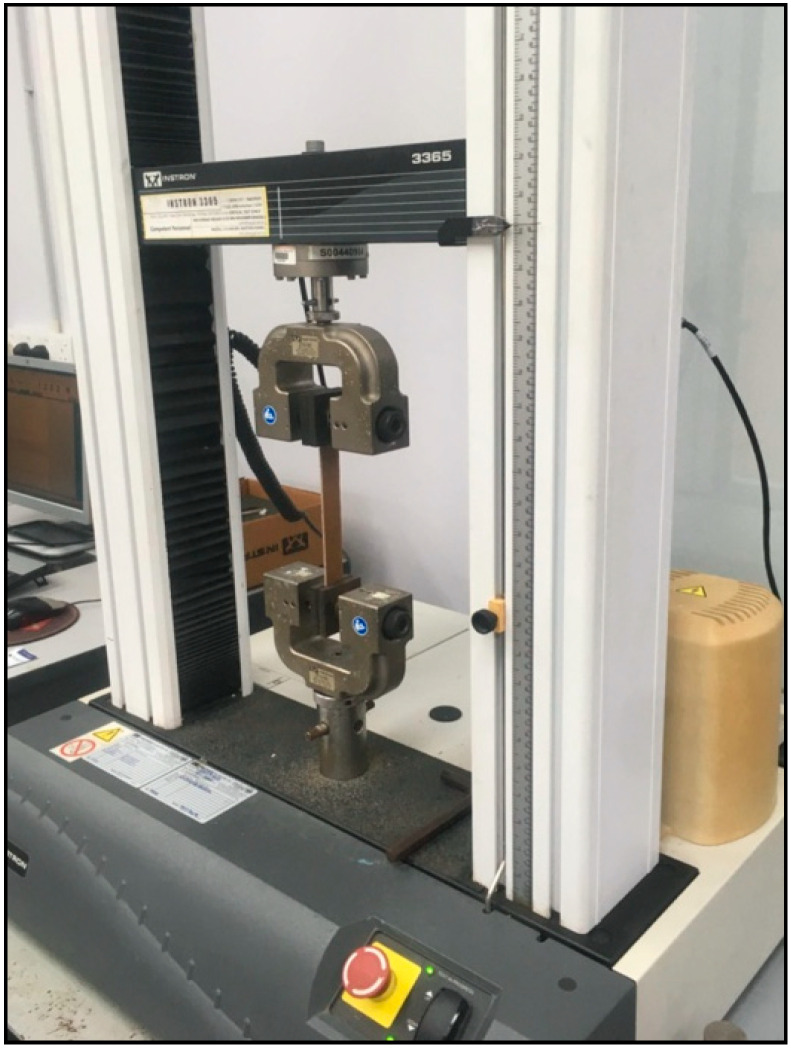
Tensile test setup using an Instron universal testing machine model 3365.

**Figure 5:**
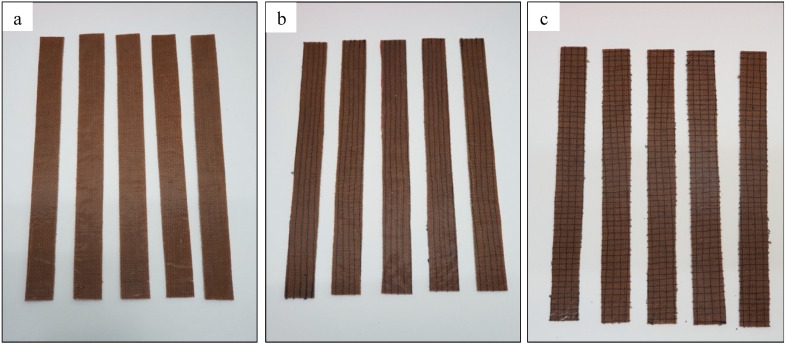
Unstitched and stitched woven kenaf tensile specimens: (**a**) (UNSTITCHED) unstitched woven kenaf-reinforced epoxy composite; (**b**) (WKS/X) silk-fiber-stitched in X-direction woven kenaf-reinforced epoxy composite; (**c**) (WKS/XY) silk-fiber-stitched in both X and Y directions woven kenaf-reinforced epoxy composite.

ASTM D3039/D3039M-14 [28] standard was taken as the reference for the tensile test. An Instron universal testing machine (model 3365 with Bluehill software, Instron, Norwood, MA, USA) with a 100-kN load capacity was used to measure the tensile mechanical properties of the unstitched and the silk-fiber-stitched woven kenaf-reinforced epoxy composites, as shown in Figure 4. The specimens were cut into rectangular shapes according to the standards, with a dimension of 250 mm length, 25 mm width, as shown in Figure 5. Five (5) specimens from each group of specimens were tested at room temperature with a head cross speed of 2 mm/min and a gauge length of 150 mm.

#### 2.3.2. Flexural Testing

**Figure 6:**
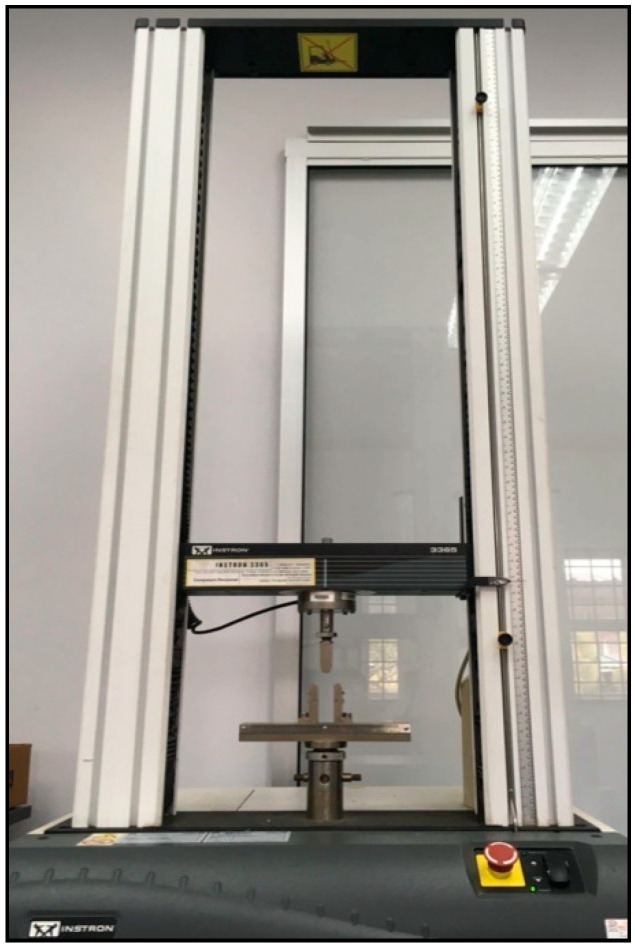
Flexural test setup using an Instron universal testing machine model 3365.

**Figure 7:**
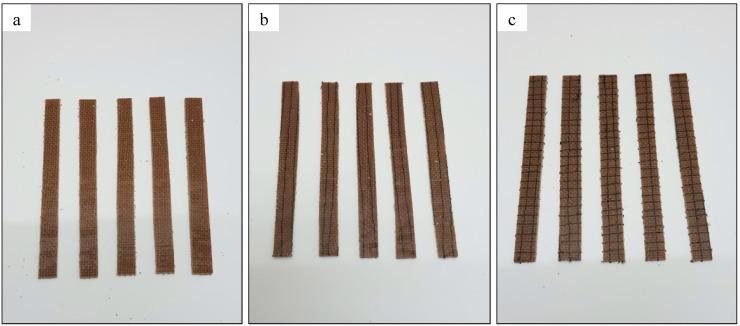
Unstitched and stitched woven kenaf flexural specimens: (**a**) (UNSTITCHED) unstitched woven kenaf-reinforced epoxy composite; (**b**) (WKS/X) silk-fiber-stitched in X-direction woven kenaf-reinforced epoxy composite; (**c**) (WKS/XY) silk-fiber-stitched in both X and Y directions woven kenaf-reinforced epoxy composite.

ASTM D790-10 [29] standard was taken as the reference for the flexural test. An Instron universal testing machine (model 3365 with Bluehill software, Instron, MA, USA) with 10-kN load capacity was used to conduct 3-point bending flexural test and to calculate the flexural properties of the unstitched and the silk-fiber-stitched woven kenaf-reinforced epoxy composites, as shown in Figure 6. The specimens were cut into rectangular shapes according to the standards, with a dimension of 127 mm length, 12.7 mm width, as shown in Figure 7. Five (5) specimens from each group of specimens were tested at room temperature, with a head cross speed of 2 mm/min. The span length was calculated using a 16:1 length to depth ratio.

#### 2.3.3. Impact Testing

**Figure 8:**
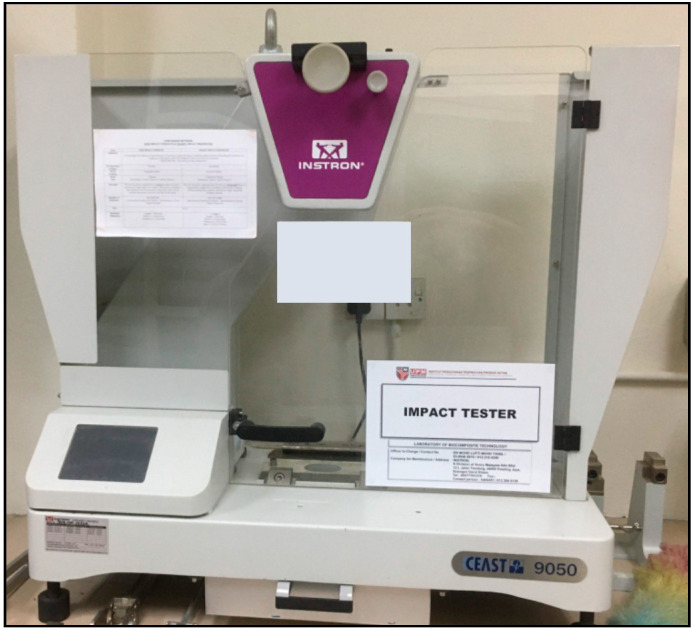
CEAST 9050 Izod impact test machine.

**Figure 9:**
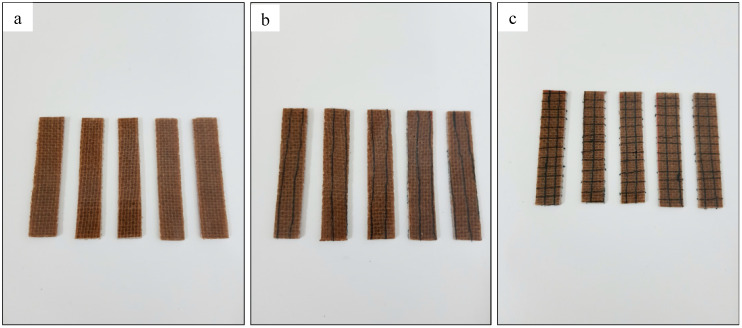
Unstitched and stitched woven kenaf impact specimens: (**a**) (UNSTITCHED) unstitched woven kenaf-reinforced epoxy composite; (**b**) (WKS/X) silk-fiber-stitched in X-direction woven kenaf-reinforced epoxy composite; (**c**) (WKS/XY) silk-fiber-stitched in both X and Y directions woven kenaf-reinforced epoxy composite.

ASTM D256-10 [30] standard was taken as the reference for the Izod impact test. An Instron CEAST 9050 testing machine was used to conduct an Izod impact test and to calculate the impact strength of the unstitched and the silk-fiber-stitched woven kenaf-reinforced epoxy composites, as shown in Figure 8. The specimens were cut into rectangular shapes according to the ASTM standards, with a dimension of 63.5 mm length, 12.7 mm width, as shown in Figure 9. Before running the test, all the specimens were notched using a notch cutter. The notched depth was 2.5 mm for all specimens. The test energy was 1 J and the test speed was 3 m/s. Eight (8) specimens from each group of specimens were tested at room temperature.

### 2.4. Composite Failure Mechanism

The failure mechanism examinations were conducted using a stereomicroscope model LEICA MS5 with five-step magnification to observe the fractured surfaces of the specimens after composite failure. The stereomicroscope analysis was carried out to investigate fiber pull-out, fiber debonding, fiber–matrix adhesion and matrix cracking.

## 3. Results and Discussion

### 3.1. Tensile Test Results

**Figure 10:**
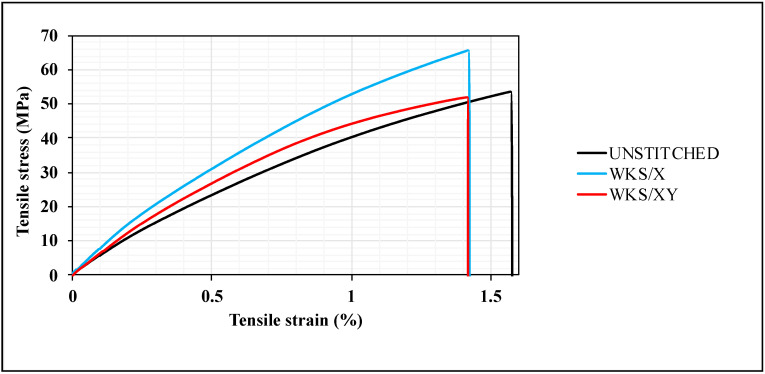
Tensile stress versus tensile strain average curves of the unstitched and silk-fiber-stitched woven kenaf composites.

The first set of mechanical properties that were investigated in this study were the tensile strength, tensile modulus and elongation to break. To start with, both the tensile strength and tensile modulus of the unstitched and silk-fiber-stitched woven kenaf composites were studied. Figure 10 shows the tensile stress versus tensile strain average curves of the unstitched and silk-fiber-stitched woven kenaf composites.

**Figure 11:**
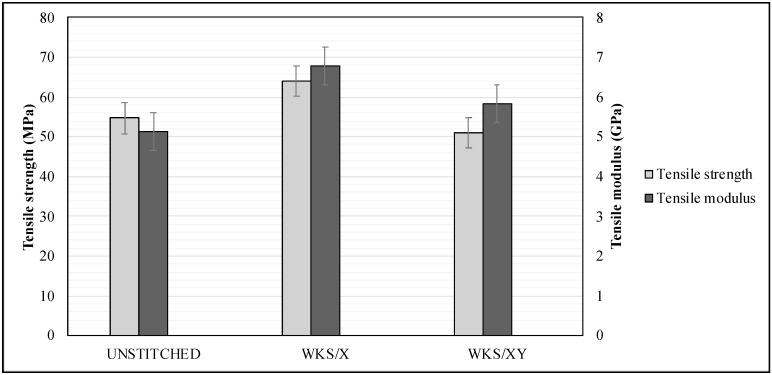
Effect of stitching density and pattern on the tensile properties of the unstitched and silk-fiber-stitched woven kenaf composites.

As shown in Figure 10, the through-thickness stitching does not significantly affect the tensile strength. This could be correlated with the structure of the plant fiber and its cell walls and this means that localized damage of the fiber cells because of stitching will not cause the failure of the whole fiber [31,32]. It is assumed that the silk-fiber-stitched specimens would have lower tensile strength compared to unstitched specimens. In general, needle insertion through the laminates during the stitching process brings some disadvantages, such as stress concentration at the stitching areas, fiber crimping and misalignment and fiber breakage. As a result, a decrease in the in-plane mechanical properties due to the stitching has been reported by many researchers [33,34,35]. However, this is not always the case. Some researchers noted an increase in both tensile strength and tensile modulus after through-thickness stitching [24]. In this study, the stitching thread did not reduce the tensile strength in the case of the WKS/X. More importantly, WKS/X stitched specimens have higher tensile strength compared to the unstitched specimens. Yaakob et al. [25] also agreed with this claim. Figure 11 shows the effect of stitching pattern and density on the tensile strength and modulus of the unstitched and silk-fiber-stitched woven kenaf composites.

It is believed that the through-thickness stitching works to prevent the formation and propagation of cracks that occur during the tensile loading, especially delamination; also, stitching helps to rearrange stress equally among the reinforcing woven kenaf [24]. Consequently, an enhancement in the tensile strength of the stitched laminates can be observed. Moreover, WKS/X was stitched parallelly to the tensile load direction (warp direction). Stitching will increase the fiber volume fraction resulting from the extra stitching thread [36]. Therefore, these specimens showed a significant increase in tensile strength. WKS/X-stitched specimens showed 17.1% and 25.6% higher tensile strength than UNSTITCHED and WKS/XY, respectively. Predictably, the higher stitching density will cause more stress concentration at the stitching areas, fiber crimping and misalignment and fiber breakage in the case of WKS/XY. Moreover, these specimens have stitching lines that go in a direction that is perpendicular to tensile loading beside the line that goes in a parallel direction to the tensile loading. The stitching lines that are transverse to the load direction (weft direction) cause crimping to the fiber that is parallel to the load direction. This crimping might also be considered as a reason that reduces the tensile strength and it also may explain why WKS/XY specimens usually fail along the stitching lines [22]. The tensile modulus value of WKS/X was 30.5% and 15% higher than UNSTITCHED and WKS/XY, respectively. By looking at Figure 11, it is clear that both stitched specimens have higher tensile modulus than the unstitched specimens.

**Figure 12:**
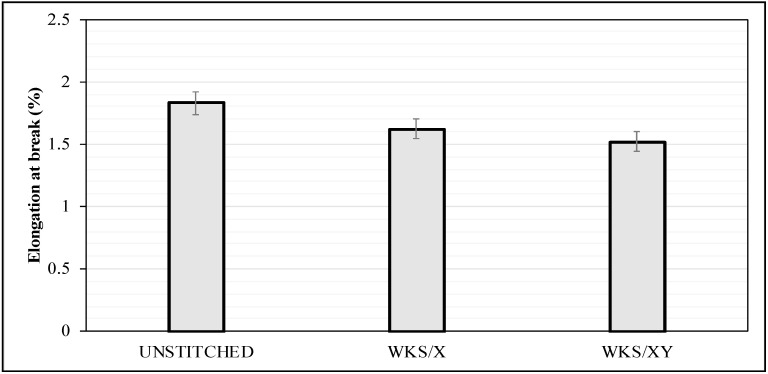
Effect of stitching density and pattern on the elongation at break of the unstitched and silk-fiber-stitched woven kenaf composites.

Elongation to break values of the unstitched and silk-fiber-stitched woven kenaf composite specimens are shown in Figure 12. It can be observed that the stitched specimens have less elongation compared to the unstitched specimens. Stitched specimens will need higher amounts of load to obtain the same elongation values due to the additional strength of the stitching thread [37]. The more densely stitched specimens of WKS/XY showed lower elongation at break values.

### 3.2. Flexural Test Results

**Figure 13:**
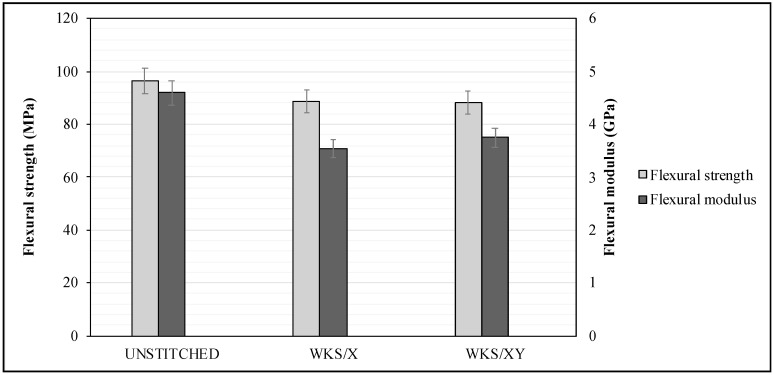
Effect of stitching density and pattern on the flexural properties of the unstitched and silk-fiber-stitched woven kenaf composites.

The flexural strength and flexural modulus were also analyzed to study the effect of the through-thickness stitching on the flexural properties of the woven kenaf-reinforced epoxy composites. Figure 13 shows the effect of stitching density and pattern on the flexural properties of the unstitched and silk-fiber-stitched woven kenaf composites. Both stitched specimens showed lower flexural strength compared to the unstitched specimens. Although stitching should have increased the interlaminar strength, which will result in enhanced flexural strength, the stress concentration introduced by the stitching process should be considered as a reason for the decrease in flexural properties.

According to [24], the interlaminar resistance of laminates plays a great role in the flexural properties in a 3-point bending test. Stitched specimens would have higher flexural strength if the stitching thread were capable of stopping interlaminar delamination [38,39]. The two stitched specimens show around the same flexural strength and this means that stitching with higher density and in the two directions, X and Y, did not reduce the flexural properties. Moreover, the specimens WKS/XY stitched in two directions showed 5.9% higher flexural modulus comparing to WKS/X-stitched specimens. The flexural strength average value of the UNSTITCHED specimens was 8.6% and 9.4% higher than the WKS/X and the WKS/XY, respectively. On the other hand, the flexural modulus average of the five specimens was 4.6 ± 0.08 GPa, 3.54 ± 0.19 GPa and 3.75 ± 0.16 GPa for UNSTITCHED, WKS/X and WKS/XY, respectively.

**Figure 14:**
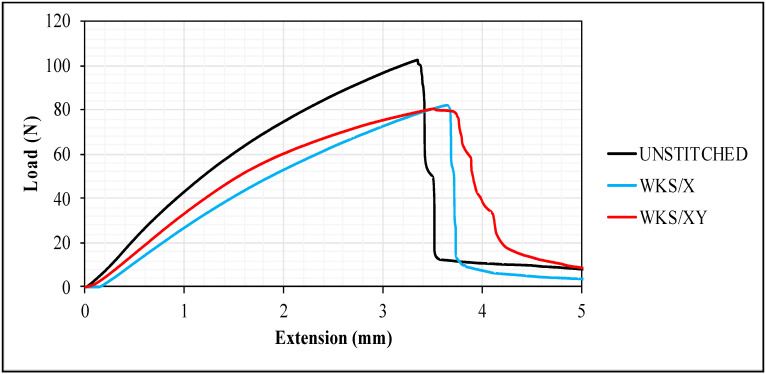
Load versus extension average curves for the unstitched and silk-fiber-stitched woven kenaf composites.

Failure processes of stitched and unstitched specimens are shown in Figure 14. By looking at the figure, it can be observed that crack growth is slower in the WKS/XY. It is believed that slower crack growth means better interlaminar strength and that stitching is able to resist intralaminar damage. However, with this improvement in the interlaminar resistance, stitching introduces stress concentration at stitching points, which is responsible for reducing the overall flexural strength of the stitched composite [24]. The failure mechanism of the WKS/X specimens is identical to the unstitched specimens, as shown in Figure 14, which could indicate that the drawbacks that come with the stitching process have created the improvement in the interlaminar resistance.

### 3.3. Impact Test Results

**Figure 15:**
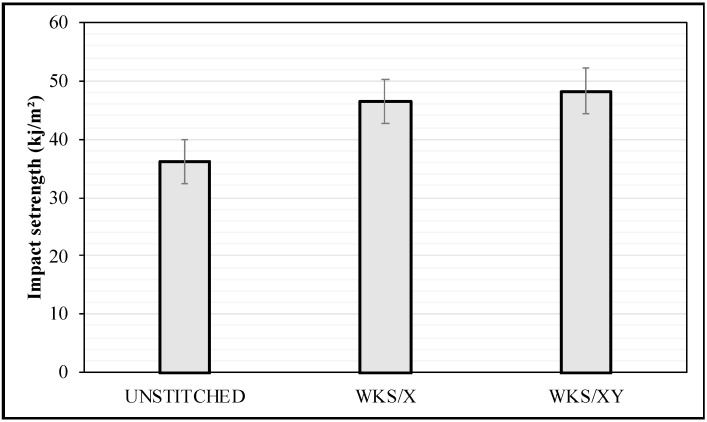
Effect of stitching density and pattern on the impact strength of the woven kenaf composite.

The impact strength average values of the unstitched and silk-fiber-stitched woven kenaf-reinforced epoxy composites at different stitch row directions and densities have been analyzed. In general, both stitched specimens show a higher impact strength. Figure 15 shows the effect of stitching pattern and density on the impact strength of stitched and unstitched woven kenaf-reinforced epoxy composite specimens. The effect of stitching on the impact strength was studied. From Figure 15, it is clear that both stitched specimens have higher impact strength compared to the unstitched specimens. The impact strength average value of the WKS/X specimens was 28% higher than the average value of the UNSTITCHED specimens. For stitched specimens, more energy is required to make the specimens fail under the same conditions and this means that stitched specimens absorb more energy. The through-thickness stitching tends to resist the propagation of the delamination and reduce its size. The crack grows between the stitching rows until it is resisted by a stitch. While the impact is considered to be a through-thickness load, the through-thickness stitches are the main load-carrying units. The Z-direction stitching threads are bridging the crack tips and absorbing more energy and this is known as the “bridging effect” [40]. Stitches work as crack suppression units and also work to distribute the stress evenly through the matrix, which will require higher loads for the crack to propagate. In contrast, the UNSTITCHED specimens suffer from pure crack propagation without any through-thickness enforcement to resist crack growth. On the other hand, the WKS/XY specimens show 33% and 3.5% higher impact strength than both the UNSTITCHED and the WKS/X specimens, respectively. Similar results were reported by [22,24]; it was found that specimens with higher stitching density have higher intralaminar toughness. This might be related to the higher stitching density or the stitching lines that go in the Y direction. The WKS/XY specimens have double stitching density as compared to the WKS/X specimens and also have stitching lines that go in the Y direction, as mentioned earlier. Thus, the crack will require more energy to overcome the interlaminar resistance that has been increased with the rise in the stitching density. More importantly, crack propagation will be resisted in the two directions X and Y and this means a greater “bridging effect”.

### 3.4. Composite Failure Mechanism

**Figure 16:**
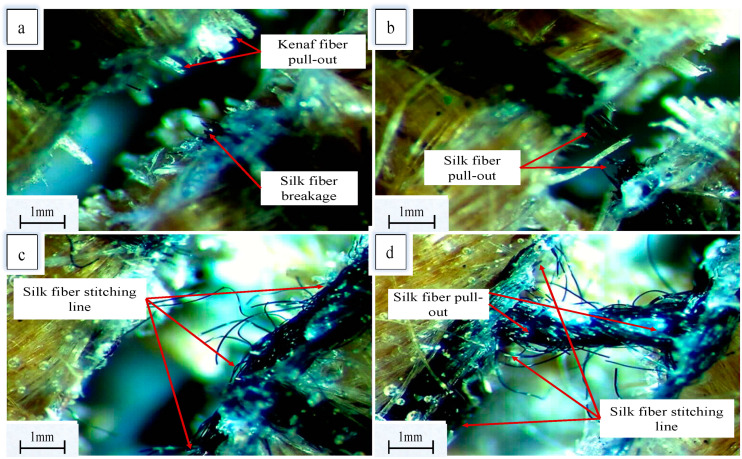
Stereomicroscope photos for the fractured surfaces: (**a**,**b**) WKS/X stitched specimens; (**c**,**d**) WKS/XY stitched specimens.

In general, the failure mechanism of the yarn that is used to stitch laminates has been proven to be controlled by yarn debonding from the polymer matrix, yarn elastic stretching, yarn pull-out and yarn breakage. Stitching yarn stretching is usually the factor that contributes to the increase in delamination resistance [41]. By looking at fracture surfaces in Figure 16, the fracture surfaces of all stitched specimens contain extra stitching thread pull-out from the kenaf composites. Besides this, silk fiber breakage can also be observed at the fractured surfaces of the stitched specimens. Silk fiber pull-out and breakage indicate the contribution of the Z-direction stitching to the interlaminar resistance [40]. Consequently, the crack will need more energy to propagate through the stitched specimens [42]. On the other hand, the unstitched specimens suffer from crack growth without any Z-binder to bridge crack tips and slow crack propagation. This might explain the higher impact resistance of the stitched specimens. Figure 16c,d show the fracture surfaces of WKS/XY-stitched specimens. It can be noticed that the failure happened along the stitching line. The reasons behind this are stress concentration at the stitching areas, fiber crimping, fiber misalignment and fiber breakage caused by needle insertion. Generally, the stitching threads work to resist the propagation of delamination and its size, which results in better impact properties.

## 4. Conclusions

In this work, 3D natural fiber-stitched composites were fabricated successfully. Both the in-plane and through-thickness mechanical properties of the unstitched and silk-fiber-stitched woven kenaf-reinforced epoxy composites have been experimentally investigated. It can be concluded that stitching the woven kenaf with silk yarn in both X-direction and X, Y directions has no significant effect on the in-plane mechanical properties of the composites. In addition, the X-direction-stitched composite has higher tensile properties compared to the unstitched composite because stitching works to distribute the applied load evenly. However, a slight decrease in the flexural strength was noticed in both stitched specimens because the stitching thread was not strong enough to stop the interlaminar delamination caused by the flexural load. From the results, it was proven that stitching did not greatly affect the tensile and flexural strength of the woven kenaf-reinforced epoxy composites. In the case of the impact strength, stitched specimens showed a significant improvement compared to the unstitched specimens. The Izod impact mechanical test results showed a significant improvement of 28% and 33% for the WKS/X and WKS/XY-stitched specimens, respectively, compared to the unstitched specimens, which means that stitching successfully improved the out-of-plane mechanical properties. The results of this research show that stitched specimens have better mechanical performance compared to unstitched specimens and that the reduction in the flexural strength is insignificant in contrast to the great improvement in the impact strength.

## Figures and Tables

**Table 1:** Summary of the stitching parameters and notation.

Specimen	Lay Up	Stitching Material	Stitching Direction	Stitch Length S_L_ mm	Stitch Row Spacing S_R_ mm	Stitch Areal Fraction (%)	Stitch Density mm^‒2^	$v_{f}\%$
**UNSTITCHED**	[0]_3_	-	-	-	-	-	-	36 ± 2
**WKS/X**	[0]_3_	Silk	X 0°	5	5	0.48	0.04	37.3 ± 2
**WKS/XY**	[0]_3_	Silk	X, Y 0°, 90°	5	5	0.96	0.08	38 ± 2
